# Rewarming: facts and myths from the neurological perspectives

**DOI:** 10.1186/cc11282

**Published:** 2012-06-07

**Authors:** Erich Schmutzhard, Marlene Fischer, Anelia Dietmann, Raimund Helbok, Gregor Broessner

**Affiliations:** 1 Department of Neurology, Neurocritical Care Unit, Medical University Hospital Innsbruck, Austria

## 

Standard operating procedures both for scientific study protocols as well as in routine daily practice stress the importance of slow rewarming after targeted temperature management/moderate therapeutic hypothermia (32 to 34°C) [1-3]. The recommended rewarming speed ranges from 0.1 to 0.4°C/hour, the former allowing a minimum of 24 hours to reach normothermia levels whereas in case of the latter the normothermic temperature levels are reached within 6 to 8 hours. It has been well known and it is widely accepted that abrupt temperature changes, with insufficient temperature control methods, cause change in energy expenditure and intracranial pressure and a negative effect onto the cerebral perfusion pressure and cerebral blood flow. In avalanche survivors the so-called afterdrop has been described to be an additional potentially dangerous condition [4,5]; hypercapnia has been shown to increase the core temperature cooling rate in snow burial victims; furthermore, shivering influences both cooling and rewarming and the cooling afterdrop [4,5].

In the rat model the speed of rewarming plays a crucial role in the development of acute lung injury in intestinal ischemia treated with therapeutic hypothermia. Reactive oxygen species have been shown to play an important role in the pathogenesis of various injuries, including brain injury and lung injury [6]. Besides this, inflammatory reaction and nitric oxide levels are clearly influenced both by therapeutic hypothermia and speed of rewarming. It might be surmised that more gradual rewarming and the addition of anti-inflammatory drugs during this period of rewarming [7,8] might add to or even enhance the neuroprotective effect of therapeutic hypothermia/targeted temperature management [9,10]. This assumption is underlined by the findings that biogenic amines that have been demonstrated to protect cells from apoptotic cell death (for example, serotonin and dopamine) protect cells against rewarming-induced active oxygen species formation and apoptosis [11]. Thus, it is correctly hypothesized that adding drugs containing these biogenic amines or releasing endogenously serotonin and dopamine might help to prevent potential negative side effects of rewarming [11].

Most of the knowledge of post-hypothermic rewarming effects has been gained in experimental settings [1,12,13]. More than 10 years ago the neuroprotective effect visualized by amyloid precursor protein positive axonal swellings, quantifying them per unit area and, thus, serving as marker of both pathophysiologic and neuroprotective effect respectively, has been shown to be reversed completely when normothermia was achieved very rapidly within 20 minutes [3,14]. The originally documented axonal protection was not only eliminated by this rapid rewarming but, in fact, the overall burden of axonal damage was dramatically increased, reaching a virtual doubling of the numbers of damaged axons seen per unit area [3]. Not only amyloid precursor protein accumulation but also neurofilament compaction was seen to be exacerbated by rapid rewarming [3].

Besides this and other neuropathological effects there is now clear evidence that cerebral microcirculation, both in traumatic brain injury and in other severe intracranial diseases, is structurally and functionally perturbed and that this perturbation can be attenuated by hypothermia followed by slow rewarming [2,15]. Similar to the situation with axonal injury, discussed above, the hypothermic protection against cerebral microcirculation impairment can be reversed or the injury even exacerbated by rapid post-hypothermic rewarming [2]. In addition, compelling evidence has been found that traumatically induced or hypoxia-induced generation of oxygen radicals significantly contributes to secondary insult onto brain cells but also damaging endothelium and smooth muscle cells [13]. When employing hypothermic intervention followed by slow rewarming, significant vascular protection was provided; in particular, the generation of oxygen radicals was reduced [14]. The protective effect of hypothermia, once again, is not only a function of time of initiating target temperature and overall duration of hypothermia. Even more importantly, all these positive effects are reversed by rapid rewarming enhancing vascular dysfunction following neuronal injury [10]. Povlishock and Wei clearly state that in the context of traumatically induced axonal damage, microvascular dysfunction, and cerebral contusion, any potentially beneficial effect of hypothermic intervention is consistently reversed and the lesion even exacerbated when post-traumatic hypothermia is followed by rapid rewarming [2]. Most likely, both direct and indirect mitochondrial perturbation together with processes mediated by free radicals influence the ensuing biology and pathophysiology. Post-hypothermic rewarming rates and the potential adverse consequences of rapid rewarming are closely observed in the field of transplantation medicine wherein hypothermic organ maintenance and rewarming are integral to successful organ viability and subsequent transplantation. Rapid rewarming is highly damaging in case of liver transplantation, most likely due to rapid ATP depletion, energy failure and oxygen radical production associated with hepatic mitochondrial damage.

Extrapolation from this body of experimental and human medical knowledge clearly indicates/allows the statement that any type of targeted temperature management/therapeutic hypothermia needs to be followed both by very slow rewarming on the one hand and maintenance of normothermia after reaching the normothermia level on the other hand [16-19]. Exactly this aspect of slow/very slow rewarming has been partially neglected in most recently published studies [19,20]; in particular, on therapeutic hypothermia in traumatic brain injury [21]. In view of the ongoing pathophysiologic processes leading to secondary neuronal damage over a period of more than 5 days post trauma, not only that the duration of therapeutic hypothermia but also the speed of rewarming (0.4°C/hour) need to be questioned and might be interpreted as the major cause of the negative study outcome.

Every researcher, when compiling a study protocol, as well as every clinician is strongly advised to accept the impact of duration of hypothermia and speed/rate of rewarming as being crucial elements both in research and clinical practice. Although it might correctly be surmised that the duration of therapeutic hypothermia is associated with the incidence and intensity of complications, the same might hold true for too short a duration and too quick a rewarming rate. Further studies are needed to evaluate various durations (1 day vs. 3 or 5 days, vs. even longer) both in traumatic brain injury but also in other neurologic diseases that trigger off secondary pathophysiological processes such as ischemic stroke (concept of penumbra), spontaneous intracerebral hemorrhage (peri-hematoma edema), traumatic brain injury or spontaneous aneurysmatic subarachnoid hemorrhage with pathophysiologic processes going on for days and even weeks after the acute ictus. In view of these considerations, prolonging therapeutic hypothermia and, in particular, slowing the rate of rewarming might be the crucial clue to achieve the best possible benefit from targeted temperature management/moderate therapeutic hypothermia without counteracting these benefits by too speedy a rewarming rate. In addition, maintaining normothermia after hypothermia and rewarming seems to be essential in order to avoid the negative rebound effects [22,23].

Finally, it needs again to be noted that a too rapid rewarming might lead to vasodilatation, thus aggravating intracranial pressure, reducing cerebral perfusion pressure and leading to a negative effect on overall neurological outcome [1,19].
